# The time is now: making the case for compassionate use/pre-approval access to new TB drugs

**DOI:** 10.5588/ijtldopen.25.0644

**Published:** 2025-12-10

**Authors:** A. Reuter, J. Stillo, S.S. Thi, I. Motta, G.B. Migliori, A. Mesic, M. Mbenga, P. Howell, L. Guglielmetti, A. Gebhard, T. DeCroo, L. D’Ambrosio, J. Furin

**Affiliations:** 1 Stellenbosch University, Stellenbosch, South Africa;; 2 Wayne State University, Detroit, MI, USA;; 3 National TB Program, Mbabane, Eswatini;; 4 Médecins Sans Frontières, Amsterdam, the Netherlands;; 5 Istituti Clinici Scientifici Maugeri, IRRCS, Tradate, Italy;; 6 KNCV, The Hague, the Netherlands;; 7 Wits Health Consortium, University of the Witwatersrand, Johannesburg, South Africa;; 8 Department of Infectious, Tropical Diseases and Microbiology, IRCCS Sacro Cuore Don Calabria Hospital, Verona, Italy;; 9 Institute for Tropical Medicine, Antwerp, Belgium;; 10 Public Health Consulting Group, Lugano, Switzerland;; 11 Harvard Medical School, Boston, MA, USA.

**Keywords:** tuberculosis, drug-resistant TB, new drugs, ethics

## Abstract

Although the majority of people with multidrug-resistant/rifampicin-resistant TB (MDR/RR-TB) can now be successfully treated with all-oral shorter regimens, rising rates of resistance to the components in these regimens are a growing global challenge. The drug development pipeline for TB has more novel compounds in phase 2 trials than ever before, and accessing these agents through systematic compassionate use programmes is an ethical and public health imperative. There is more than a decade of successful experience with compassionate use for MDR/RR-TB, resulting in benefits for people with MDR/RR-TB, novel chemical entity developers, providers, and programmes. Although there are some arguments for delaying access to the new TB drugs in the pipeline, we present reasons why such access should be an urgent priority for all TB stakeholders.

Almost half a million people become sick from multidrug-resistant/rifampicin-resistant TB (MDR/RR-TB) annually and the WHO recommends that a majority of individuals with these types of TB be treated with all-oral shorter regimens.^1^ Most of these regimens contain bedaquiline, linezolid, a third-generation fluoroquinolone, clofazimine, and/or a nitroimidazole such as delamanid or pretomanid. When a strain of *Mycobacterium tuberculosis* (*M.tb*) has resistance to one or more of these drugs or they cannot be given due to intolerance, treatment is more challenging and requires longer, more toxic regimens. Some forms of resistance are associated with a low chance of cure.^2,3^ Strains of *M.tb* with resistance to bedaquiline, linezolid, and/or a nitroimidazole – sometimes referred to as having expanded resistance – are a growing problem globally. Depending on the populations assessed, baseline rates as high as 7% have been reported.^4^ In people whose cultures are still positive after 4 months of treatment, resistance rates as high as 47% have been reported.^5^ While prior treatment with these drugs is a key risk factor for resistance, ongoing transmission is also occurring.^6^ Urgent action is needed to both cure the individuals with strains of *M.tb* that have expanded resistance but also to stop the spread of these types of TB.^2^ Experience has shown that when highly resistant strains of TB are not effectively treated, they can cause major, deadly outbreaks of disease.^7^ When designing regimens for people with strains of TB that have expanded resistance, there are limited effective options, especially given the cross-resistance that occurs between bedaquiline and clofazimine.^5^ To address this growing concern, providers, programmes, and impacted communities have called for early access to newer TB drugs, which are being developed at a pace not seen before in TB.^8^ There are multiple new TB drugs currently in the pipeline^9^; these include novel drugs that have completed phase 2b trials with encouraging interim results.^10^ For market-based reasons and because these trials are easier to enrol, most of these drugs are being assessed for treatment shortening for pan-susceptible TB.^9^

Pre-approval access programmes (sometimes called ‘clinical access,’ ‘expanded access,’ or ‘compassionate use’ [CU] programmes) are one way in which newer TB drugs could be accessed in a systematic way to support people with MDR/RR-TB with expanded resistance.^11^ Although the definitions of these terms vary slightly depending on the context,^12^ here we will refer to all such pre-approval access as ‘CU programmes’. The TB community has more than a decade of successful experience with pre-approval access programmes for bedaquiline and delamanid.^13^ Historically, these programmes had robust components that upheld WHO principles for CU of new TB drugs.^14^ They provided life-saving treatment for a disease of public health significance while also ensuring patient safety, preventing further emerging drug resistance and upholding ethical principles such as patient autonomy and dignity. Such components include: 1) clear indications for drug use, with requests reviewed by independent clinical expert panels and formal informed consent obtained from the patient; 2) core principles for regimen design including drug susceptibility testing and regimen design with backbone therapy/companion drugs; 3) standardised practices/protocols for monitoring and ongoing clinical management, including support from expert clinicians; 4) data collection and reporting practices for key stakeholders; 5) meaningful collaboration with national and international bodies to monitor safety, outcomes, and acquired drug resistance; and 6) shared funding mechanisms to support equitable use.^15^ Not only did these programmes provide needed care for people with MDR/RR-TB with no other therapeutic options, but they also helped advance the knowledge base for these drugs, especially in populations that were not being enrolled in clinical trials.^16^ This in turn led to more rapid implementation in these countries due to the availability of real-world data. In addition to the guidelines from the WHO,^14^ civil society and many stringent regulatory agencies provide a clear framework for CU of new TB drugs.^17,18^

There are multiple examples of successful CU programmes in MDR/RR-TB, but perhaps one of the earliest and most illustrative occurred at a few treatment centres in France, and notably at the Bligny Hospital (Briis-sous-Forges), with bedaquiline.^13^ This programme was started in 2011 – after the bedaquiline phase 2 trial data were available but prior to bedaquiline conditional approval by stringent regulatory agencies. A robust CU programme was established following the principles of the French National Agency for the Safety of Medicine and Health Products (ANSM), which are similar to those stated earlier in this editorial. Through this programme, 45 individuals received bedaquiline over a 3-year period. Of these patients, 98% had culture conversion.^15^ These experiences with the bedaquiline CU programme helped address important clinical questions and supported the development of pivotal phase 3 trials.^9^

Despite the positive experience with pre-approval use of bedaquiline and delamanid,^19,20^ some novel chemical entity developers (NCEDs) may be reluctant to provide early access to their drugs through such programmes. In discussions on advancing pre-approval access programmes over the past 12 months, the authors of this article have encountered several misconceptions and misunderstandings. We describe these in Table and explain why such issues should not delay CU/pre-approval access.

**Table.** Potential reasons for delaying compassionate use (CU) programmes and responses supporting access.

Potential reasons for delaying CU access to new TB drugs	Response to support accelerating access
There is limited safety data, especially for longer treatment durations	Full safety profiles of drugs are often not established until they have been received by thousands of individuals, so a balance must be taken between risk of the disease and risks from drug side effects. Unless there is a valid reason to expect cumulative safety risk with longer duration (i.e., aminoglycosides), longer durations can be considered based on a risk–benefit assessment.
There are limited efficacy data, especially for the drugs as single agents since they are usually tested in combination.	With the exception of 14-day early bactericidal activity trials, TB drugs are usually tested in combination with single drug efficacy inferred from non-clinical studies, an approach that has been accepted by regulators. No new drug will be given as a single effective agent in CU programmes. Because there are so many combinations of TB drugs possible, CU programmes present a valuable way to gather safety and efficacy data on these drug combinations.
Drugs should only be made available for CU after phase 3 trials	Ethical principles, stringent regulatory agencies, and global public health guidance endorse beginning CU programmes after phase 2 data support potential efficacy and safety.^20^
Patients and providers are ‘desperate’ and not able to assess the risks and benefits objectively	Systematic approaches to TB CU have been in place for over a decade which are rigorous and focus on protecting the rights of patients through counselling and informed consent while supporting them to be active bearers of agency. TB service providers are professionals with an ethical duty to provide the best possible care for individuals seeking their services. CU will only be provided as part of rigorous programmes that are in line with existing frameworks on CU.
There is potential cross-resistance with existing drugs	There is a valid concern for drugs within the same class (i.e., the oxazolidinones), unless data suggest this resistance can be overcome (i.e., sorfequiline [TBAJ-876] and bedaquiline). For drugs with resistance mediated by the *Rv0678* mutation/efflux pump, examinations of minimum inhibitory concentrations may be helpful in supporting use.^21,22^ As novel chemical entity developers (NCEDs) develop drug susceptibility testing for the newer agents, the technology can be transferred to laboratories where CU is taking place to assess drug resistance.
There is no method for assessing susceptibility to the CU agent(s), and therefore drugs susceptibility testing cannot be done at baseline or monitored during treatment	The lag time between a drug completing phase 2 trials and methods for assessing susceptibility need to be shortened. CU programmes, however, cannot wait for drug susceptibly testing to be available, as unfortunately such testing may not be available even after a drug has been through phase 3 trials. Simultaneous, accelerated work to establish susceptibility testing to new TB drugs should be part of pre-approval access programmes.
Negative experiences with CU can contribute to a drug not being authorised by regulatory agencies	Reviews from other disease areas and from TB show that CU access has not been shown to risk regulatory approval and may, in fact, contribute to data used to approve a drug.^22^ Adequately managed CU may help programmes gain experience with newer drugs so that providers have experience and can help lead later rollout of these agents for wider populations.^23^
Drugs should not be made available as single agents but rather wait until multiple new drugs can be combined in a single new regimen	Some individuals may require only a single new agent added to a strong, effective backbone regimen and should have access to this option based on expert recommendations. At the same time, others will need combinations of new drugs, which must also be made available through CU. Access to single agents should not be delayed while awaiting the rollout of multiple drugs, provided they are combined with an adequate backbone; however, equal urgency is required to ensure that patients can also access combinations of novel agents, as these often represent their best chance of cure.
Decisions about CU should be made solely by NCEDs, including deciding who should receive the drug	NCEDs should work in partnership with expert clinical providers and impacted communities and individuals with expanded resistance to allow for joint decision making. Clinical expert committees (which include impacted community members) should be able to operate independently to determine eligibility for CU.
Research should be done instead of CU	Ethical principles and global public health guidance establish that research and CU programmes are both needed and complementary to one another. Some people needing new drugs may not be able to enrol in trials.^24^ Furthermore, CU programmes can help ensure people do not feel pressured into participating in research as the only way to access a new drug. CU is also a way for NCEDs/researchers to invest in communities that participate in their research.

## CONCLUSION

Despite significant progress in the diagnosis and treatment of MDR/RR-TB, strains of *M.tb* with expanded resistance pose a mounting global threat. Innovative partnerships between NCEDs, national TB programmes, researchers, clinical providers, and affected communities have successfully supported CU access to bedaquiline and delamanid for more than a decade and could form the basis of ongoing collaboration to provide CU access to new anti-TB drugs. We urge NCEDs and other stakeholders to examine current delays in CU, re-evaluate any false precepts, and draw on the experiences of existing frameworks to strengthen work with stakeholders to ensure a human rights–based approach to CU access. CU programmes are not only beneficial for the reasons described here but are also a moral and ethical imperative, especially for diseases that have a public health risk. By providing treatment for otherwise incurable forms of TB, we can ensure scientific progress benefits not just a few individuals in trials but entire communities, upholding the right to health and scientific progress for all.
